# Supplementary material for: The Relationship between Suicidality and Socio-Demographic Variables, Physical Disorders, and Psychiatric Disorders: Results from the Singapore Mental Health Study 2016
Source: Int J Environ Res Public Health. 2021 Apr 20;18(8):4365. doi: 10.3390/ijerph18084365 (PMC8074258; doi:10.3390/ijerph18084365)
Supplement: Supplementary file 1 [file ijerph-18-04365-s001.zip › ijerph-1137009-supplementary.pdf]

Supplementary Material 1
Suicidality Module of WHO-CIDI V21.1.4

SUICIDALITY (SD)

*SD1. INTERVIEWER CHECKPOINT:

RESPONDENT IS ABLE TO READ............................1
ALL OTHERS..2 **GO TO *SD15**

---

*SD2. (RB, PG 20) Three experiences are listed in your booklet on page 20 labeled A, B, and C. Did experience A ever happen to you ?

INTERVIEWER: EXPERIENCE A IS 'YOU SERIOUSLY THOUGHT ABOUT COMMITTING SUICIDE'

YES 1
NO.......................................5 **GO TO *SR1, NEXT SECTION**
DON'T KNOW...................8 **GO TO *SR1, NEXT SECTION**
REFUSED ....9 **GO TO *SR1, NEXT SECTION**

*SD2a. How old were you the first time this happened?

__________ YEARS OLD

DON'T KNOW 998
REFUSED 999

---

*SD3. Did Experience A happen to you at any time in the past 12 months?

YES....................................1 **GO TO *SD4**
NO 5
DON'T KNOW...................8
REFUSED...........................9

*SD3a. How old were you the last time this experience happened to you?

__________ YEARS OLD

DON'T KNOW........................ 998
REFUSED 999

***SD4.** (RB, PG 20) Now look at the second of the three experiences on the list, Experience B. Did experience B ever happen to you?

INTERVIEWER: EXPERIENCE B IS 'YOU MADE A PLAN FOR COMMITTING SUICIDE'

YES 1
NO.......................................5 **GO TO *SD6**
DON'T KNOW...................8 **GO TO *SD6**
REFUSED ....9 **GO TO *SD6**

*SD4a. How old were you the first time this happened?

__________ YEARS OLD

DON'T KNOW........................ 998

REFUSED 999

*SD5. Did Experience B happen to you at any time in the past 12 months?

YES.....................................1 **GO TO *SD6**
NO 5
DON'T KNOW...................8
REFUSED...........................9

*SD5a. How old were you the <u>last</u> time this experience happened to you?

__________ YEARS OLD

DON'T KNOW...................... 998
REFUSED 999

***SD6.** (RB, PG 20) Now look at the third of the three experiences on the list, Experience C. Did experience C <u>ever</u> happen to you ?

INTERVIEWER: EXPERIENCE C IS 'YOU ATTEMPTED SUICIDE'

YES 1
NO.......................................5 **GO TO *SR1, NEXT SECTION**
DON'T KNOW...................8 **GO TO *SR1, NEXT SECTION**
REFUSED ....9 **GO TO *SR1, NEXT SECTION**

*SD6a. How many times did Experience C ever happen to you in your lifetime?

________ NUMBER OF TIMES

DON'T KNOW................. 998
REFUSED 999

---

*SD7. INTERVIEWER CHECKPOINT: (SEE ***SD6a**)

***SD6a** EQUALS '1' .... 1 **GO TO *SD10**
ALL OTHERS.. 2

---

*SD8. How old were you the <u>first time</u>?

__________ YEARS OLD

DON'T KNOW 998
REFUSED...................................999

*SD9. (RB, PG 21) There are three statements numbered 1, 2, and 3 on page 21 in your booklet. Which of these three statements best describes your situation when Experience C happened to you the first time -- 1, 2, or 3?

I MADE A SERIOUS ATTEMPT TO KILL MYSELF AND IT WAS ONLY LUCK THAT I DID NOT SUCCEED 1

I TRIED TO KILL MYSELF, BUT KNEW THAT THE METHOD WAS NOT FOOL-PROOF.. 2

MY ATTEMPT WAS A CRY FOR HELP. I DID NOT INTEND TO DIE 3

DON'T KNOW 8
REFUSED.. 9

---

*SD10. Did Experience C happen to you in the past 12 months?

YES...1 **GO TO *SD11**
NO 5
DON'T KNOW 8
REFUSED.. 9

*SD10a. How old were you (when/the last time) experience C happened to you?

__________ YEARS OLD **GO TO *SD14**

DON'T KNOW ....998 **GO TO *SD14**
REFUSED ....999 **GO TO *SD14**

***SD11.** Did it result in an injury or poisoning?

YES.....................................1
NO.......................................5 **GO TO *SD14**
DON'T KNOW ....8 **GO TO *SD14**
REFUSED ....9 **GO TO *SD14**

---

*SD12. Did it require medical attention?

YES.....................................1
NO.......................................5 **GO TO *SD14**
DON'T KNOW ....8 **GO TO *SD14**
REFUSED ....9 **GO TO *SD14**

---

*SD13. Did it require overnight hospitalization?

YES.....................................1
NO 5
DON'T KNOW 8
REFUSED...........................9

***SD14.** (RB, PG 21) Looking at page 21 in your booklet, which of the three statements best describes your situation when Experience C happened to you (the last time) – 1, 2, or 3?

I MADE A SERIOUS ATTEMPT TO KILL MYSELF AND IT WAS ONLY LUCK THAT I DID NOT SUCCEED 1

I TRIED TO KILL MYSELF, BUT KNEW THAT THE METHOD WAS NOT FOOL-PROOF 2

MY ATTEMPT WAS A CRY FOR HELP. I DID NOT INTEND TO DIE 3

DON'T KNOW 8

REFUSED 9

---

*SD14.1. INTERVIEWER CHECKPOINT: (SEE ***SD10**)

***SD10** EQUALS '1' 1
ALL OTHERS .... 2 **GO TO *SR1, NEXT SECTION**

---

*SD14.2. (RB, PG 22) Please look at page 22 in your booklet. Which method did you use (when/the last time) Experience C happened to you? (Just give me the letter.)

A. GUN 1
B. RAZOR, KNIFE OR OTHER SHARP INSTRUMENT 2
C. OVERDOSE OF PRESCRIPTION MEDICATIONS 3
D. OVERDOSE OF OVER-THE-COUNTER MEDICATIONS 4
E. OVERDOSE OF OTHER DRUG (E.G. HEROIN, CRACK, ALCOHOL) 5
F. POISONING (E.G. CARBON MONOXIDE, RAT POISON) 6
G. HANGING, STRANGULATION, SUFFOCATION 7
H. DROWNING 8
I. JUMPING FROM HIGH PLACES 9
J. MOTOR VEHICLE CRASH 10
K. OTHER (PLEASE DESCRIBE) 11

____

____

DON'T KNOW 98
REFUSED 99

GO TO *SR1, NEXT SECTION

***SD15.** The next few questions are about thoughts of hurting yourself. Have you ever seriously thought about committing suicide?

YES 1
NO .... 5 **GO TO *SR1, NEXT SECTION**
DON'T KNOW .... 8 **GO TO *SR1, NEXT SECTION**
REFUSED .... 9 **GO TO *SR1, NEXT SECTION**

*SD15a. How old were you the first time this happened?

__________ YEARS OLD

DON'T KNOW 998
REFUSED 999

*SD16. Have you seriously thought about committing suicide at any time in the past 12 months?

YES....................................1 **GO TO *SD17**
NO 5
DON'T KNOW...................8
REFUSED...........................9

*SD16a. How old were you the <u>last</u> time this experience happened to you?

__________ YEARS OLD

DON'T KNOW..................................998
REFUSED 999

***SD17.** Have you ever made a <u>plan</u> for committing suicide?

YES.....................................1
NO......................................5 **GO TO *SD19**
DON'T KNOW...................8 **GO TO *SD19**
REFUSED ....9 **GO TO *SD19**

*SD17a. How old were you the <u>first</u> time this happened?

__________ YEARS OLD

DON'T KNOW..........................998
REFUSED 999

---

*SD18. Did you make a plan for committing suicide at any time in the past 12 months?

YES....................................1 **GO TO *SD19**
NO 5
DON'T KNOW...................8
REFUSED...........................9

*SD18a. How old were you the <u>last</u> time this experience happened to you?

__________ YEARS OLD

DON'T KNOW..................................998
REFUSED 999

***SD19.** Have you ever attempted suicide?

YES.....................................1
NO......................................5 **GO TO *SR1, NEXT SECTION**
DON'T KNOW...................8 **GO TO *SR1, NEXT SECTION**
REFUSED ....9 **GO TO *SR1, NEXT SECTION**

*SD19a. How many times have you attempted suicide in your lifetime?

________ NUMBER OF TIMES

DON'T KNOW................. 998
REFUSED 999

*SD20. INTERVIEWER CHECKPOINT (SEE ***SD19a**):

***SD19a** EQUALS '1' .... 1 **GO TO *SD23**
ALL OTHERS 2

---

*SD21. How old were you the first time?

__________ YEARS OLD

DON'T KNOW 998
REFUSED..................................999

---

*SD22. There are three statements I will read out loud. Please tell me which of these three statements best describes your situation when you attempted suicide the first time – one, two, or three?

"One, I made a serious attempt to kill myself and it was only luck that I did not succeed."
"Two, I tried to kill myself, but knew that the method was not fool-proof."
"Three, my attempt was a cry for help, I did not intend to die."

I MADE A SERIOUS ATTEMPT TO KILL MYSELF AND IT WAS ONLY LUCK THAT I DID NOT SUCCEED 1

I TRIED TO KILL MYSELF, BUT KNEW THAT THE METHOD WAS NOT FOOL-PROOF... 2

MY ATTEMPT WAS A CRY FOR HELP. I DID NOT INTEND TO DIE 3

DON'T KNOW 8

REFUSED.. 9

***SD23.** Have you attempted suicide in the past 12 months?

YES..1 **GO TO *SD24**
NO 5
DON'T KNOW 8
REFUSED.. 9

*SD23a. How old were you (when/the last time) you attempted suicide?

__________ YEARS OLD **GO TO *SD27**

DON'T KNOW..................998 **GO TO *SD27**
REFUSED ....999 **GO TO *SD27**

***SD24.** Did it result in an injury or poisoning?

YES.....................................1
NO.......................................5 **GO TO *SD27**
DON'T KNOW..................8 **GO TO *SD27**
REFUSED ....9 **GO TO *SD27**

*SD25. Did it require medical attention?

YES....................................1
NO......................................5 **GO TO *SD27**
DON'T KNOW.................8 **GO TO *SD27**
REFUSED ....9 **GO TO *SD27**

---

*SD26. Did it require overnight hospitalization?

YES....................................1
NO 5
DON'T KNOW 8
REFUSED..........................9

***SD27.** There are three statements I will read out loud. Please tell me which of these three statements best describes your situation when you attempted suicide (the <u>last</u> time) – one, two, or three?

"One, I made a serious attempt to kill myself and it was only luck that I did not succeed."
"Two, I tried to kill myself, but knew that the method was not fool-proof."
"Three, my attempt was a cry for help, I did not intend to die."

I MADE A SERIOUS ATTEMPT TO KILL MYSELF AND IT WAS ONLY LUCK THAT I DID NOT SUCCEED 1

I TRIED TO KILL MYSELF, BUT KNEW THAT THE METHOD WAS NOT FOOL-PROOF.. 2

MY ATTEMPT WAS A CRY FOR HELP. I DID NOT INTEND TO DIE 3

DON'T KNOW 8

REFUSED... 9

---

*SD28. INTERVIEWER CHECKPOINT: (SEE ***SD23**)

***SD23** EQUALS '1' 1
ALL OTHERS.. 2 **GO TO *SR1, NEXT SECTION**

---

*SD29. Which method did you use (when/the last time) you attempted to commit suicide?

A. GUN 1
B. RAZOR, KNIFE OR OTHER SHARP INSTRUMENT... 2
C. OVERDOSE OF PRESCRIPTION MEDICATIONS..3
D. OVERDOSE OF OVER-THE-COUNTER MEDICATIONS.. 4
E. OVERDOSE OF OTHER DRUG (E.G. HEROIN, CRACK, ALCOHOL)..................... 5
F. POISONING (E.G. CARBON MONOXIDE, RAT POISON) 6
G. HANGING, STRANGULATION, SUFFOCATION.. 7
H. DROWNING...8
I. JUMPING FROM HIGH PLACES.. 9
J. MOTOR VEHICLE CRASH.. 10
K. OTHER (PLEASE DESCRIBE).. 11

____

____

DON'T KNOW 98
REFUSED.. 99

GO TO *SR1, NEXT SECTION
